# The Potential for Absolute Temperature Imaging Based on Brain Metabolites Using an FID‐Shifting Approach in Gradient Echo Planar Spectroscopic Imaging (GREPSI)

**DOI:** 10.1002/mrm.70487

**Published:** 2026-06-23

**Authors:** Dennis L. Parker, Henrik Odéen, Peyton Wong, Seong‐Eun Kim, Wesley Judd, Duane D. Blatter

**Affiliations:** ^1^ Utah Center for Advanced Imaging Research, Department of Radiology and Imaging Sciences University of Utah Salt Lake City Utah USA

## Abstract

**Purpose:**

To implement and evaluate an efficient method for absolute temperature measurement using a gradient echo implementation of the echo planar spectroscopic imaging (EPSI) sequence.

**Methods:**

The gradient echo EPSI sequence (GREPSI) utilizes an oscillating readout gradient together with continuous signal readout after slab selective excitation. The metabolite peaks are separated from the large water signal using a novel free induction decay (FID) time shifting method. SNR comparisons were made between spectra acquired with strong, weak, and no water suppression on a brain phantom with physiological metabolite concentrations. Measurements were made using no water suppression with the phantom at discrete temperatures between 22°C and 42°C. GREPSI was also compared with single voxel spectroscopy (SVS) and chemical shift imaging (CSI) pulse sequences. Finally, a healthy volunteer was scanned.

**Results:**

The FID shifting method allowed removal of the water spectrum and recognition of metabolite peak locations independent of water suppression. The standard deviation of the temperature measured at room temperature was 0.31°C, independent of water suppression, respectively. The absolute temperature measurements closely matched the corresponding water‐bath temperature with accuracy within 2% at 42°C and precision on the order of 0.2°C using either NAA, Cre, or Cho as a reference. The in vivo temperature images of a normal volunteer were in the expected normal range.

**Conclusion:**

This study indicates that accurate and precise temperature measurements can be obtained with GREPSI in a scan time on the order of 2–5 min, for an 8‐slice slab, depending on the FOV imaged.

## 1 Introduction

### 1.1 Background

The knowledge of temperature and temperature distribution within the brain is critical to understand the state of healthy and diseased brain, to follow the progression of brain tissue after acute injury, and to monitor critically important thermal interventions such as hypothermia, hyperthermia, and thermal ablation. Brain temperature is known to respond to intracranial infections [1], stroke [2, 3, 4], and tumors [5]. Post‐operative infection rates after intercranial surgery are roughly 2%–6% [6, 7, 8] but early infections can be difficult to recognize as a patient may not have a fever or abnormal serum markers [7, 9]. It may be possible to detect the infection by measuring temperature distributions with high spatial accuracy. Subtle variations in intracranial temperature may indicate early inflammatory responses or abscess formation.

Hypothermia lowers brain temperature to reduce metabolism and continued damage while the brain recovers from a traumatic brain injury (TBI) [10] or stroke [11, 12, 13, 14]. Hyperthermia increases blood flow and tumor oxygenation to improve the efficacy of chemo‐ or radiation‐therapy. These temperature changes are small (3°C–7°C) and absolute temperature must be known accurately over an extended treatment time interval (often more than 30 min) to ensure that the brain reaches the target temperature and prevents overcooling or overheating [15].

Because even small temperature changes can affect neuronal function [16, 17], it is crucial to be able to measure absolute brain temperature within an accuracy and precision of better than 1.0°C [18] and for some applications higher accuracy within 0.2°C to 0.5°C may be needed [19, 20]. For example, a decrease of 0.3°C can be clinically relevant in conditions like stroke and TBI [18]. While this precision can be achieved using the proton resonance frequency shift method, its accuracy is often unknown since it can only measure temperature change.

Magnetic resonance spectroscopy (MRS) methods for measuring absolute temperature [21, 22, 23, 24] are based on the difference between the water proton resonant frequency, which changes with temperature, and the frequencies of protons on biological molecules such as N‐acetyl aspartate (NAA), creatine (Cre), and choline (Cho), which do not change with temperature. Absolute temperature in the brain was initially measured using single voxel spectroscopy (SVS) methods [16, 19, 20, 25, 26, 27, 28, 29, 30, 31, 32]. Because of the very low signal from the metabolite peaks, SVS typically requires large single voxels (∼1–8 cm^3^) and multiple averages to achieve adequate signal to noise ratio (SNR). To improve efficiency, the multiple averages in SVS can be spatially encoded to allow acquisition of spectra from multiple voxels, a method referred to as chemical shift imaging (CSI) [2, 33, 34, 35] or MR spectroscopic imaging (MRSI).

While CSI methods are more efficient than SVS, they are still very slow and the low metabolite concentrations result in very small signals allowing acquisition of a small number of large voxels (e.g., 8 × 8 × 1 voxels with voxel dimensions > 1 cm^3^). Substantially increased speed and resolution in MRSI can be achieved by interleaving spatial and spectral encoding in a method referred to as echoplanar spectroscopic imaging (EPSI) [36, 37, 38, 39, 40, 41, 42]. EPSI has been used to measure absolute temperature using NAA as the reference metabolite in multiple studies [43, 44, 45, 46, 47, 48, 49, 50, 51, 52, 53, 54]. Even with improved efficiency, EPSI measurements can require 15 to 30 min for a volume covering the brain [50, 55].

Despite the slow speed, spectroscopic methods have been used to measure absolute brain temperature distributions for a variety of applications (diseases, injuries, and interventions) [20, 56]. Normal brain temperature has been shown to vary spatially and across individuals (from 36.1°C to 40.9°C in healthy adults) and regionally within the same subject by about 2.4°C [17]. In a population of TBI patients (*n* = 114), the temperature varied between subjects from 32.6°C to 42.3°C [17]. TBI can disable the brain's ability to selectively self‐cool, resulting in catastrophic hyperthermia [2]. Temperature may also be dysregulated in fever and stroke [27]. Regional temperature variations can be caused by spatial variations in blood flow, water content [17], and metabolic rate [2] and have been measured in normal and infarcted brain [57] using whole brain EPSI [43, 45] during active conductive head cooling [58]. Diurnal variations can be on the order of 0.6°C to 1.2°C [17].

Despite their potential utility, MRSI and EPSI methods still lack sufficient speed or spatial resolution to be viable for clinical applications. In nearly all CSI and EPSI implementations, out‐of‐volume inversion or saturation pulses are used to localize the volume, and refocusing pulses are used to give an echo‐time (TE) when all metabolites and water have zero phase, making it possible to correct the phase of the complex spectrum to that point in time. With these pulses, EPSI can require tens of minutes for small, low‐resolution tissue volumes. Methods with increased spatial and temporal resolution must be developed before absolute temperature can be effectively used in disease characterization and treatment monitoring.

To improve the efficiency of EPSI methods, we implemented a Gradient Echo EPSI (GREPSI) sequence, which has no SE pulses to rephase the signals and may not require water, lipid, or out‐of‐volume saturation pulses. Prior multi‐echo gradient echo spectroscopy sequences have been developed for characterizing fat in breast MRI as well as measuring temperature using fat or ethylene glycol [59, 60, 61, 62, 63]. These sequences are not adequate for detecting the small metabolite peaks in the brain.

Our implementation of GREPSI allowed 2D or 3D acquisition, a selectable number of gradient lobes and a short repetition time (TR) giving a trade‐off between number of lobes (frequency resolution) and acquisition speed (TR). Water, fat, and/or out of volume saturation were optional. Lacking a refocusing pulse, the linear phase correction was based on the time offset from the excitation pulse and the frequency offset from water (which was given zero phase). In this paper, we describe our implementation of the GREPSI sequence and the unique processing methods that enable metabolite peak detection without water suppression. Although the metabolite peak amplitudes are more than an order of magnitude smaller than the dispersive component of the water spectrum at the metabolite peak frequencies, the phase of the metabolite peaks relative to the water spectrum varies with FID start time. By varying the effective FID start time, our algorithms separate the water and metabolite signals, such that the metabolite peaks can be accurately detected and used as a temperature reference relative to the water peak.

The accuracy of metabolite peak detection was evaluated with and without water suppression.

## 2 Methods

### 2.1 Pulse Sequence

**FIGURE 1:**
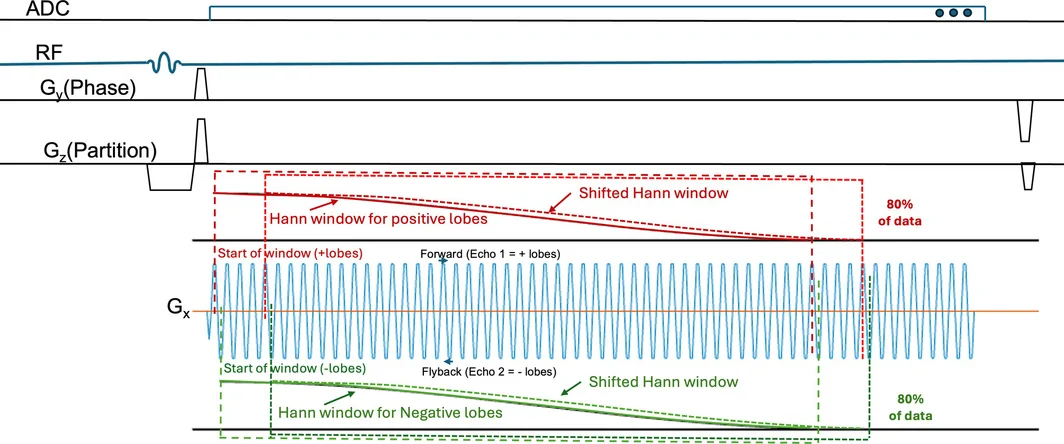
3D MR GREPSI pulse sequence implemented using a 3D segmented echo planar imaging (EPI) readout with dual‐echo acquisition (Echo 1 and Echo 2). Each k‐space segment is acquired with an oscillating readout gradient pattern, alternating between forward and flyback trajectories. Bottom (G_x_) is an illustrative diagram showing the Hann window weighting used for spectra reconstruction from the echoes in the GREPSI echo train. The width of the Hann window can be adjusted based upon the relative SNR in the measured spectrum. To generate data with multiple FID delay times, the Hann window is shifted as shown.

GREPSI is a spoiled GRE pulse sequence with an oscillating readout gradient as shown graphically in Figure 1. The duration of each readout lobe was 0.56 ms (period of 1.12 ms for the positive and negative lobe pair). To avoid ghosting artifacts, we separated the data by positive and negative lobes, giving a time between lobes (of same polarity) of 1.12 ms, or a frequency bandwidth of 1/1.12 ms = 893 Hz. This bandwidth was sufficient to cover the ∼350 Hz separation of NAA from water at 3 T as well as the ∼420 Hz separation of lipid peaks. With a sampling dwell time of 5 μs, 112 readout samples including oversampling were acquired during each lobe. With oversampling (doubling the field of view in the readout direction), 64 samples were acquired during the flat part of the gradient, with 24 samples acquired during the ramp up and 24 during the ramp down time. During spatial reconstruction, the 24 ramp points were gridded into 12 equally spaced k‐space points with the same spacing as the central 64 points. In our sequence, 576 lobes were acquired after each excitation giving 288 positive and 288 negative lobes. To improve SNR and eliminate Gibbs artifact from the spectrum, we applied a half Hann window (one at the first readout point and covering 80% of the data) effectively giving a spectral resolution of 7.76 Hz. The FID was zero‐filled interpolated [64] from 288 to 4096 points before Fourier transformation, giving a spectral spacing of 0.22 Hz.

All scans were performed at 3 T (PrismaFIT, Siemens Healthineers, Erlangen, Germany) using the 16 head channels of the Siemens (OEM) 20 channel head and neck coil.

### 2.2 Phantom Scans

The GREPSI sequence was used to acquire multiple 2D scans of a brain metabolite phantom (Model 2 152 220, General Electric Company, Milwaukee, WI), containing physiological concentrations of NAA, Creatine, and Choline. GREPSI parameters used include TR/TE = 600/1.67 ms, FA = 40°, FOV = 200 × 200 × 20 mm^3^, voxel dimensions = 4.55 × 6.25 × 20 mm^3^, acquisition matrix = 44 × 32 × 1 (after overscan removal) with image reconstruction interpolated to 64 × 64 × 1, for an in‐plane voxel spacing of 3.125 mm, Table 1. Scans were repeated with strong, weak, and no water suppression. For non‐heating phantom scans one measurement average was acquired (DAQ time = 19.2 s). For the study with the phantom heated to multiple temperatures, measurements were obtained with strong, weak, and no water suppression, each with three measurement averages (58 s). For each scan, a total of 576 × 16 images were reconstructed (one image for each of the 576 gradient lobes and for each of the 16 receiver coil channels) using a fast Fourier transform. All data processing was performed in MATLAB (Mathworks, Natick MA).

**TABLE 1:** GREPSI scan parameters.

Number of lobes	576 (288 positive/288 negative)	
Time between lobes	0.56 ms (1.12 ms between pos lobes)	
Sampling dwell time	5 μs	
Samples per lobe	56 (112 with oversampling)	
Frequency bandwidth	1000 ms/1.12 ms = 893 Hz	
Frequency resolution	893 Hz/288 = 3.10 Hz	
80% of lobes	893 Hz/(0.8 × 288) = 3.88 Hz	
Adding Hann window	893/(0.5 × 0.8 × 288) = 7.76 Hz	
Spectral interpolation	288 to 4096 gives 0.218 Hz spacing	
Gradient echo time (TE)	2.46, 3.02, 3.58, 4.14, 4.70, …	
	**Phantom**	**Human**
Acquisition FOV	200 × 200 × 20 mm^3^	200 × 200 × 40 mm^3^
Matrix	56 × 32 × 1	56 × 32 × 8
Voxel Dimension	3.57 × 6.25 × 20 mm^3^ = 0.45 cm^3^	3.57 × 6.25 × 5 mm^3^ = 0.112 cm^3^
Repetition time (TR)	600 ms	328 ms
Acquisition time	19.2 s	84 s (< 1.5 min)

### 2.3 Temperature Calibrations Scan

To calibrate the temperature dependence of the water to metabolite spectral spacing the phantom was placed in a water bath heated to 5 different temperatures: 22°C, 27°C, 32°C, 37°C, 42°C. The phantom was left in the water bath at each temperature for at least 24 h to equilibrate. Scans (same parameters as above) were then performed at the 5 different temperatures. The spectral peak spacings between water, NAA, Cre, and Cho were converted to temperature using slopes published by Maudsley: [50]

(1) $$T_{\mathrm{NAA}}=-102.76\cdot \Delta_{H_{2}O-\mathrm{NAA}}+312.1{}^{\circ}C$$

(2) $$T_{\mathrm{Cre}}=-102.61\cdot \Delta_{H_{2}O-\mathrm{Cre}}+207.4{}^{\circ}C$$

(3) $$T_{\mathrm{Cho}}=-103.06\cdot \Delta_{H_{2}O-\mathrm{Cho}}+189.8{}^{\circ}C$$

These intercept values were slightly increased from those published by Maudsley [50] (+310.5°C, +206.1°C, and +187.7°C for NAA, Cre, and Cho, respectively). To compare GREPSI, SVS, and CSI temperature measurements, these studies were repeated at 22°C, 26°C, 30°C, and 36°C. At each temperature, the acquisition order was GREPSI, CSI, SVS, and GREPSI again. SVS and CSI were performed with stock vendor‐supplied pulse sequences (Siemens) using parameters given in Table 2.

**TABLE 2:** SVS_SE (press) CSI_SE (press).

Sampling dwell time	833.35 μs	833.35 μs
Frequency bandwidth	1200 Hz	1200 Hz
Frequency resolution	1.17 Hz	1.17 Hz
Vector Size	1024	1024
Echo Time (TE)	30 ms	30 ms
Acquisition FOV	20 × 20 × 20 mm^3^	160 × 160 × 15 mm^3^
Matrix	1	16 × 16 (2D)
Voxel Dimension	20 × 20 × 20 mm^3^	10 × 10 × 15 mm^3^
Average	20	1
Repetition time (TR)	2000 ms	1700 ms
Acquisition time	50 s	4:20 min

### 2.4 In Vivo Volunteer Scans

With IRB approved informed consent, one healthy volunteer was scanned using MPRAGE for image volume localization and the GREPSI sequence with all parameters the same, except setup as a 3D acquisition with 8 slices covering a 200 × 200 × 40 mm^3^ volume. With no water suppression, TR was reduced from 600 ms to 328 ms, Table 1. A few months later the same human volunteer was scanned with six repetitions of GREPSI with the same imaging parameters. For comparison, a single CSI_se 32 × 32 voxel image covering a 200 × 200 × 10 mm FOV was obtained.

### 2.5 Image Reconstruction and Spectral Processing for Coil Combination

For all experiments, the 2D or 3D images for each receiver coil and for each gradient lobe were first reconstructed by the 2D or 3D spatial Fourier transform. After the spatial transform, the images were separated by gradient polarity (positive and negative gradient lobes).

A phase shift, $\vartheta_{c}\left(\vec{x}\right)$, was determined for each coil, *c*, for each voxel, $\vec{x}$, for each gradient direction to allow for coil combination. For the phantom study with strong water signal, the phase was determined from the phase of the first point, $t_{1}$, of the FID:

(4) $$\vartheta_{c}\left(\vec{x},t_{1}\right)=\text{angle}\left(m_{c}\left(\vec{x},t_{1}\right)\right)$$

when a strong lipid signal was present in some voxels, the FID signals for all voxels in all images for all coils were converted to the corresponding spectrum with Hann windowed zero filled interpolation and $\vartheta_{c}\left(\vec{x},t_{1}\right)$ was determined as the phase of the water peak. To implement an optimal coil combination and maintain an accurate spectral phase in the complex signal, the complex value (magnitude and phase) at the water peak of the first image in the FID was used as the complex coil sensitivity, $b_{c}\left(\vec{x}\right)=m_{c}\left(\vec{x},t_{1}\right)$, and the combined signal was then obtained as [65, 66]:

(5) $$m^{\prime }\left(\vec{x},t\right)=\sum_{c^{\prime }}\;\sum_{c}m_{c}\left(\vec{x},t\right)\Psi_{c,c^{\prime }}^{-1}b_{c^{\prime }}^{*}\left(\vec{x}\right)$$

The noise covariance matrix, $\Psi_{c,c^{\prime }}$, between receiver channels was calculated using regions in the image data with no signal. This is possible for all of our data because the image acquisition is performed with oversampling in the readout direction. When reconstructed, the overscan region consists of noise only data, unless there are artifacts. Fortunately, artifacts are rare and can be recognized.

### 2.6 Post Coil‐Combine Spectral Processing

After coil combination, the frequency spectrum for each voxel was determined separately for the positive and negative lobe images by first multiplying the FID by a half‐Hann window of the selected width (80% of the 288 positive or negative lobes), Figure 1, and then zero filling to 4096 points before Fourier transformation to obtain the spectrum at each voxel:

(6) $$s\left(\vec{x},f_{k}\right)=\sum_{j=0}^{N_{\mathrm{zfi}}-1}h\left(\frac{j}{N_{\mathrm{FID}}}\right)m^{\prime }\left(\vec{x},t_{j}\right)e^{-2\pi if_{k}t_{j}}$$

where *N*_zfi_ = 4096, *N*_FID_ = 0.8 × 288 and:

(7) $$h\left(u\right)=\frac{1}{2}\left(\cos\left(\pi u\right)+1\right)0\leq u<1$$

h(u)=0otherwise

### 2.7 Field Flattening B_0_ Correction

After coil combination, each voxel spectrum was frequency‐aligned by shifting the water resonance to a common reference frequency (0 Hz). This step effectively removed any resonant frequency variations due to B_0_ field variations across the imaging volume. As a result, subsequent frequency shifts of the metabolite peaks reflect temperature‐dependent chemical shift changes rather than underlying spatial B_0_ inhomogeneity.

### 2.8 Metabolite Detection

The dependence of spectrum phase on acquisition delay is covered in detail in the Supporting Information. In summary, without water suppression, the dispersive (imaginary) part of the water spectrum is sufficiently broad that it dominates the peak amplitudes of the metabolites of interest. If the water signal exhibits mono‐exponential decay, Equation (6) can be written:

(8) $$\begin{array}{cc} s_{w}\left(\vec{x},f_{k}\right) & =m_{w}^{\prime }\left(\vec{x},t_{0}\right)\sum_{j=0}^{N_{\mathrm{zfi}}-1}h\left(\frac{j}{N_{\mathrm{FID}}}\right)e^{-\frac{t_{j}}{T_{2w}}}\;e^{-2\pi if_{k}t_{j}}\\ & =m_{w}^{\prime }\left(\vec{x},t_{0}\right)\;g\left(T_{2w},f_{k}\right) \end{array}$$

Equation (8) can apply to any metabolite, *x*, with *w* replaced by *x*. If $N_{\mathrm{FID}}$ is large enough, $g\left(T_{2},f_{k}\right)$ can be approximated as an integral, giving:

(9) $$g\left(T_{2},f_{k}\right)=\int_{0}^{\infty }\mathrm{dt}\;e^{-\frac{t}{T_{2}}}\;e^{-2\text{πift}}=\frac{T_{2}}{1+4\pi^{2}f^{2}T_{2}^{2}}-\frac{i2\pi fT_{2}^{2}}{1+4\pi^{2}f^{2}T_{2}^{2}}$$

We define $R_{y}$ to be the ratio of the metabolite (y) peak to the amplitude of the water spectrum at the resonant frequency of the metabolite:

(10) $$R_{y}=\frac{C_{y}}{C_{w}}2\pi ∆fT_{2y}$$

For NAA, where $\frac{C_{y}}{C_{w}}∼0.009/42$, ∆f∼330Hz, and $T_{2\mathrm{NAA}}∼0.100\;\text{to}\;0.200\;s$, the ratio $R_{y}$ will be about 0.05 to 0.10, making detection of the NAA peak difficult without water suppression.

Without knowledge of the phase of the NAA signal relative to the water signal at that point, it is difficult to locate the very weak NAA signal.

### 2.9 Detection and Removal of Water Signal

In the Supporting Information, we show that the difference between the phase of the metabolite peak and the phase of the water spectrum at the location of the metabolite peak varies linearly with the sampling delay, $t_{0}=t_{l}$ in Equation (8):

(11) $$\varphi_{w,l}\left(f_{y}\right)-\varphi_{y,l}\left(f_{y}\right)=2\pi \left(f_{y}-f_{w}\right)t_{l}+\varphi_{g}$$

We investigated this effect using different FID start delays, $t_{l}$. This is similar to acquisition with multiple TE's used to reduce the effects of eddy currents [67]. Rather than re‐acquisition with different time delays, the different time delays were directly accomplished by shifting the half Hann window down the echo‐train, Figure 1, and setting to zero all measurements earlier in time than the center of the Hann window:

(12) $$s_{l}\left(\vec{x},f_{k}\right)=\sum_{j=0}^{N_{\mathrm{zfi}}-1}h\left(\frac{j-l}{N_{\mathrm{FID}}}\right)m^{\prime }\left(\vec{x},t_{j}\right)e^{-2\pi if_{k}t_{j}}$$

For each image voxel, we created 20 shifted spectra and observed that the phase of the metabolite peaks varied relative to the water spectrum. By averaging all 20 shifted spectra, the metabolite signals and any other off‐resonant signals were effectively removed from the spectrum. The averaged spectrum was then subtracted from the 20 shifted spectra, removing all but a small residual water signal that varied with delay time. This small residual signal was removed by subtracting a low order (2nd order) fit to the spectra in the metabolite frequency region.

### 2.10 Metabolite Peak Location

After complete water signal removal, the metabolite peaks were visible in the magnitude of the complex spectra. A two‐step process was used to accurately locate the metabolite peaks. First, the metabolite peaks were located in a spatially averaged spectrum with high SNR that was created by averaging the magnitude of the water‐removed spectra over a large volume of the brain. A linear phase based upon the time from excitation to the start of signal readout was used to correct each of the 20 shifted spectra for each image voxel. The NAA peak in each voxel was then found from the real component of the linear‐phase‐corrected spectrum within a search window of ±8°C centered around the location of the average peak. The Cre and Cho peaks were found in a ±4°C search window centered around their known offsets from the NAA peak. In the case of ablation procedures, this interval could be widened. This strategy reduced peak misidentification and improved spatial coherence of the temperature maps.

### 2.11 Statistics

To evaluate the relative precision obtained in the phantom studies, the mean and standard deviation of the measured temperature were evaluated by pooling all data in regions where the NAA and water peaks were both detected. The evaluation was performed comparing measurements by region and then pooling all data for each type of water suppression. The difference in mean temperature and standard deviation was assessed as well as the ratio of variances.

## 3 Results

**FIGURE 2:**
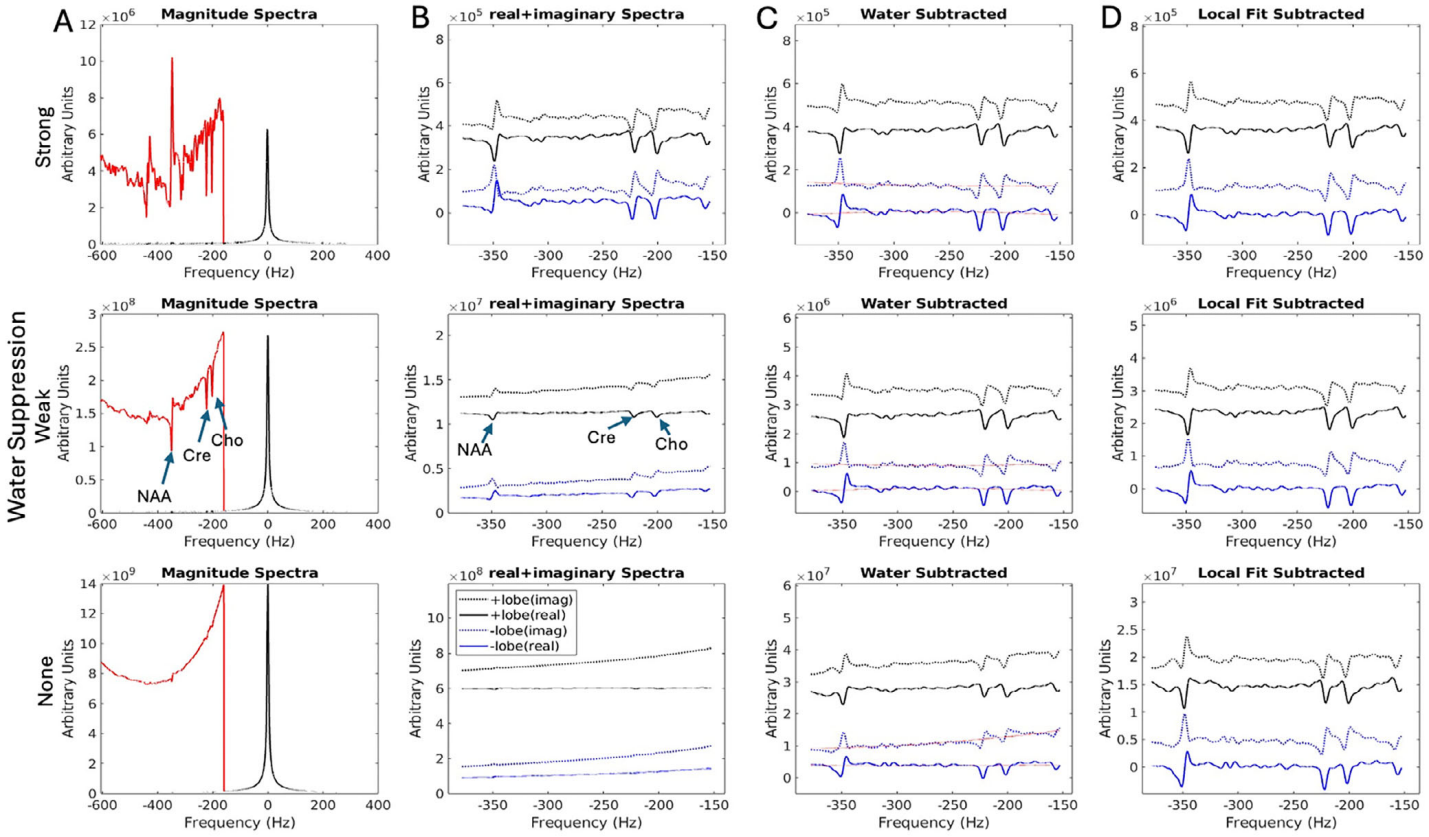
Spectra from a central voxel in the GREPSI image of the brain phantom showing the effects of strong, weak, and no water suppression. (A) Are the magnitude spectra for the positive gradient lobes with the lower frequency scaled and overlayed in red to show the peak structures encoded in the signal. (B) The real and imaginary spectra for the positive lobes (+lobe) and the negative lobes (−lobe). (C) The spectra in (B) after the average signal (presumably water signal) has been removed. A 2nd order least‐squares fit applied to the water‐subtracted spectra is shown by the red line. (D) The spectra in (C) after a local fit to the spectra has been subtracted—The effect is most visible in the non‐water‐suppressed spectrum (bottom row).

Representative spectra for the three water suppression settings (strong, weak, and no water suppression) are shown in Figure 2. Because of the uniformity of the phantom, the spectrum at each voxel is identical, except for noise and any distortions in the line shape due to eddy currents and local field inhomogeneity, and therefore only the spectrum for a single central voxel is shown. In column (A), the magnitude of the spectrum is shown—with the first half scaled so that the midpoint has about the same magnitude as the water peak. The magnitude spectrum appears to have small positive or negative contributions from the metabolites in the phantom. The small contributions of the metabolites are also seen in the real and imaginary spectra of column (B). Here, the contributions are most clearly seen with strong water suppression, less well seen with weak water suppression, and barely visible with no water suppression. The phase difference of the metabolite signals between the positive and negative gradient lobes is due to the difference in time delay from the excitation pulse to the start of the positive and negative lobe image sequences. By shifting the Hann window as shown in Figure 1 and Equation (12), we investigated the effect of the timing of the first echo on the relative phase of the spectra. The Hann window was shifted by 0 to 19 lobe positions to generate a series of 20 spectra from each gradient lobe polarity with different delays. The resulting 20 spectra were averaged to remove most of the non‐water signal, and the average “water” signal was subtracted from the original complex spectrum to yield the corrected spectrum shown in column (C).

(13) $$s_{l,\mathrm{cor}}\left(\vec{x},f_{k}\right)=s_{l}\left(\vec{x},f_{k}\right)-{\left\langle s_{l}\left(\vec{x},f_{k}\right)\right\rangle }_{l}$$

To correct for a residual bias due to changes in the magnitude of the water peak for the 20 shifted spectra, a 2nd order least squares fit (red lines in the bottom spectra in column (C)) is applied to all spectra for all voxels. In column (D) the low order (2nd order) fit to the spectra has been subtracted and the spectra are nearly identical, regardless of the water suppression used. For display, the spectra for (B) through (D) have been spread vertically. Because the phase of the complex spectrum has been adjusted such that the water peak has zero phase (zeroth order phase correction), the complex spectra show the characteristics of a Lorentzian with a broad imaginary water signal.

**FIGURE 3:**
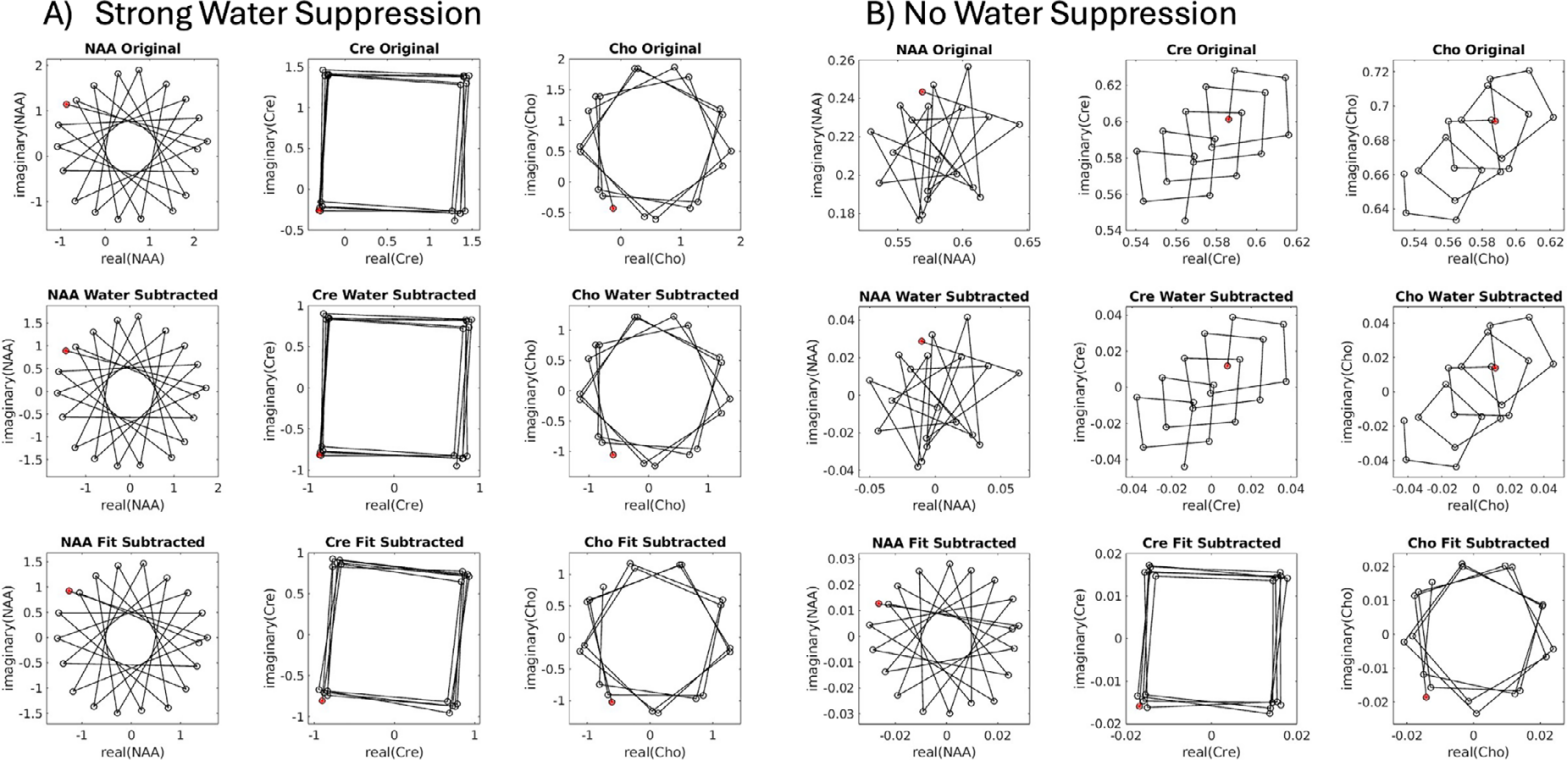
Complex signal from a single central voxel at the NAA, Cre, and Cho peaks plotted as imaginary versus real as a function of the effective signal delay from the RF excitation for strong water suppression (A) and no water suppression (B). The top row is from the original measured spectrum at the position of the metabolite peaks. The middle row is after subtraction of the average of the spectrum, presumed to be close to the water signal value. The bottom row is after subtracting a low order (2nd order) fit to the spectrum. The point with the red asterisk is the point from the Hann window center on the first lobe with no signal delay, and the subsequent points are from application of the Hann window delayed by increments of one gradient lobe (1.12 ms).

In Figure 3 and Figure S1, the real vs. imaginary values of the spectra for NAA, Cre, and Cho are plotted for all 20 Hann window shifts for the spectra obtained from the positive gradient lobes with strong, weak, and no water suppression. The red asterisk gives the real and imaginary point with zero shift, and the points for subsequent shifts are found by following the line from that point. The top row plots values from the original spectra, the middle row plots values after removing the average (assumed “water”) spectrum, and the bottom row is after subtraction of the low order (2nd order) fit. In each case the peaks are centered around a complex point that is close to zero after removal of the 2nd order fit. In Figure S2, these plots are given for weak water suppression for both gradient directions.

Figure S3 plots the spectra over the range of the metabolites with the successive Hann window shifts with the real below and the imaginary above. In this case, zero order phase correction has been performed for the water (S3a), NAA (S3b), Creatine (S3c), and choline (S3d) peaks.

Figure S4 provides temperature images that are computed from the difference between the water peak and the metabolite peaks for the cases of strong, weak, and no water suppression. Measurements for positive and negative gradient lobes are plotted as well as the average. A 2nd set of phantom measurements was obtained with the same acquisition parameters and each acquisition repeated 3 times. The mean and standard deviation of the temperature measurements were then obtained within the 4 quadrants of each phantom, and also in the center and periphery of each image. In comparing the mean and standard deviation between regions, no significant difference was observed. However, comparing the mean and standard deviation of the temperature between the different types of water suppression gave:

*T*_ave_ = 22.05 ± 0.31°C (*n* = 5298 samples, strong water suppression).

*T*_ave_ = 22.19 ± 0.31°C (*n* = 5487 samples, weak water suppression).

*T*_ave_ = 22.18 ± 0.31°C (*n* = 5433 samples, no water suppression).

Although the mean temperatures between suppression types were significantly different, the difference was very small and could likely have come from a slow warming of the phantom.

**FIGURE 4:**
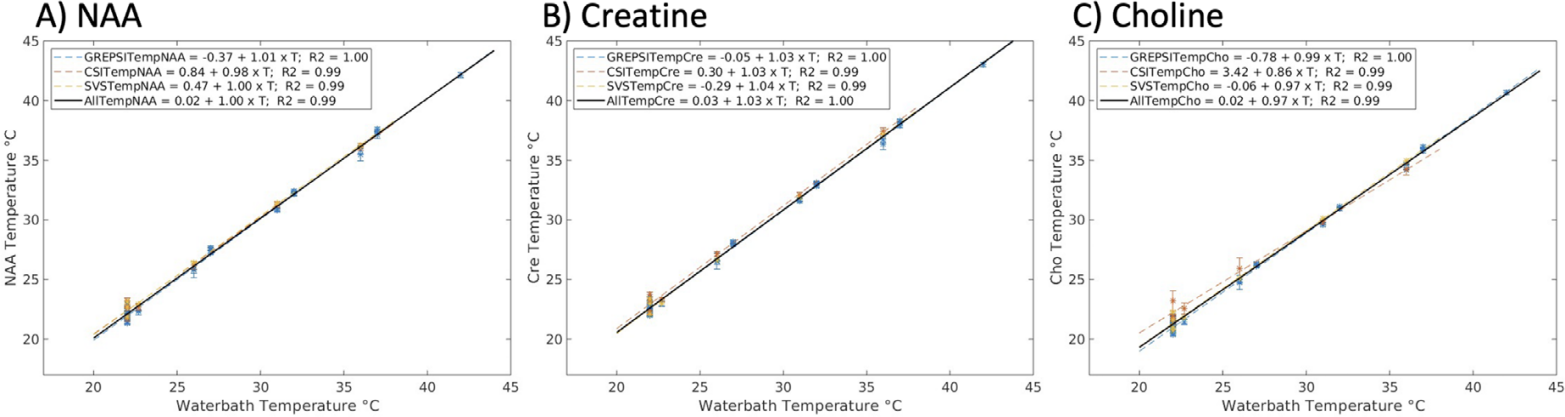
Temperatures using GREPSI images with no water suppression and each of the 3 metabolites as reference. These use the temperature versus ppm change in frequency between the water and NAA peak and the calibration values published by Maudsley with a small change (+1.6°C, +1.3°C and +2.1°C for NAA, Cre, and Cho respectively) in the offset temperature.

In Figure 4 line plots from the temperature calibration experiment are shown. In this case the temperatures were calculated from the water/metabolite frequency difference using the slopes published by Maudsley [50] and his offsets increased by +1.6°C, +1.3°C and +2.1°C for NAA, Cre, and Cho respectively. With those values, the linear regression of the measured temperatures to the water bath temperatures yields slopes very close to unity, offsets close to zero and *R* ^2^ > 0.99 for all three metabolites with no water suppression.

**FIGURE 5:**
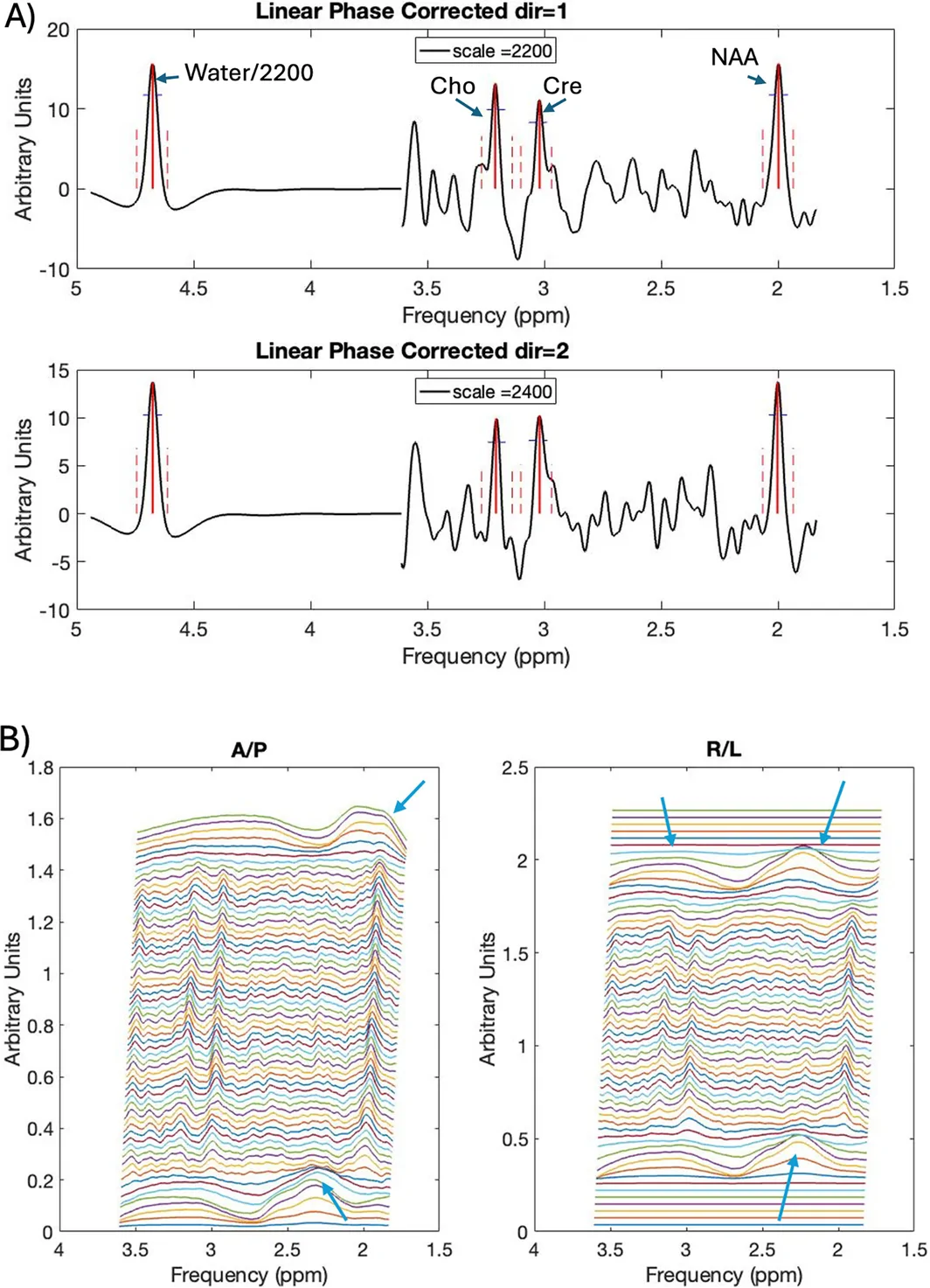
Spectra obtained by applying a linear phase shift to each of the water correct spectra of the 20 FID shifts and then averaging the 20 phase‐corrected spectra for each gradient polarity. (A) The spectra for a single central voxel at location [3, 32] in the 3D reconstructed GREPSI imaging volume. The search interval is shown by the vertical dashed lines. The peak location is defined as the midpoint of the ¾ maximum points indicated by the blue segments. For display, the left spectrum is divided by 2200 and 2400 for the two gradient directions. (B) The real spectra for the positive gradient directions for 21 voxels along a line in the A/P direction centered around the central voxel. Blue arrows indicate what may be signals from lipids in the skull.

**FIGURE 6:**
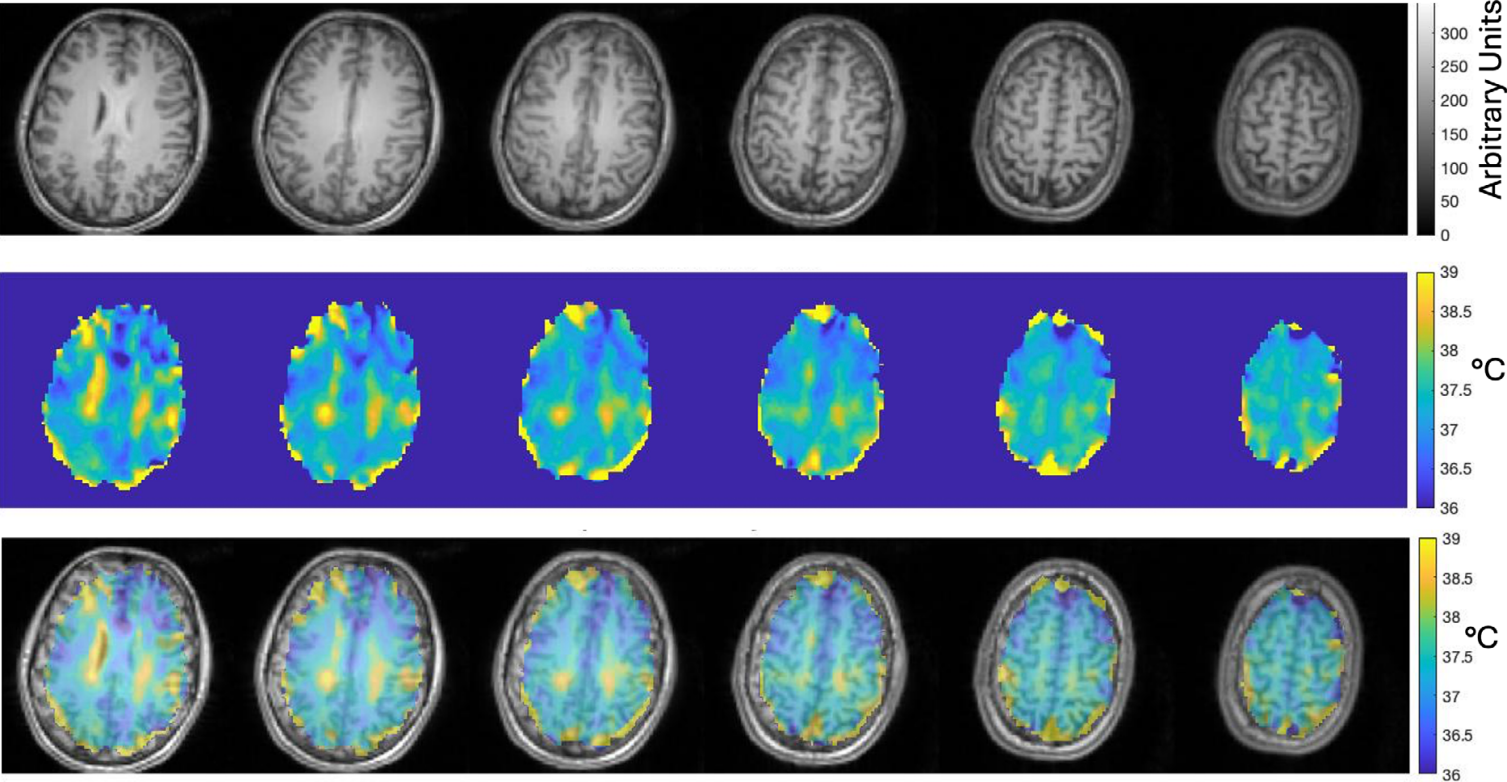
3D GREPSI Temperatures from human volunteer scan using no water suppression. Acquisition and processing parameters are described in the text. Calibration values from the phantom study were used.

**FIGURE 7:**
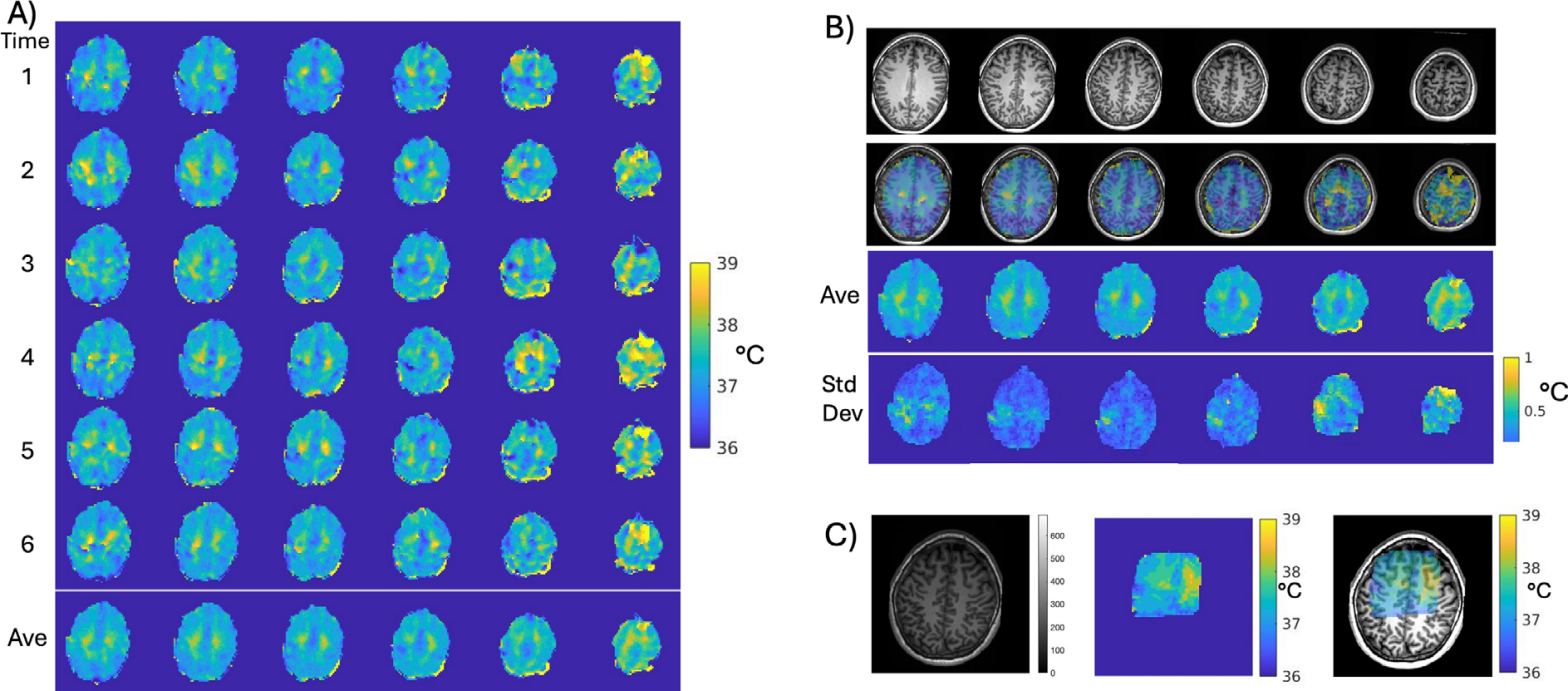
Temperature images obtained from the same volunteer as Figure 6. (A) Six sequential 8‐slice GREPSI acquisitions showing the 6 central temperature images of each temporal acquisition. (B) MPRAGE images showing the locations of the 6 temperature slices displayed; the temperatures from the 4th time overlayed on the MPRAGE images; The average and then standard deviation over time of these temperatures (SD = 0.29°, 0.24°, 0.23°, 0.28°, 0.37°, 0.53°). (C) (left) MPRAGE image from center of the 6 MPRAGE images. (middle) CSI_se Temperature image obtained with the CSI_se sequence. (right) the CSI temperature image overlayed on the MPRAGE image.

Results from the human in vivo scan are shown in Figures 5, 6, 7, S5, S6. Figure 5a shows the real spectrum from a single central voxel in the brain for both gradient directions after linear phase correction. All of the processing steps described in the methods were used and the peaks were located by first finding the NAA peak, refining it in the search window shown, and then finding the Cre and Cho peaks given their known offset from the NAA peak as shown. Figure 5b shows the spectra from a column of voxels through the center of the brain in the A/P direction and another line of voxels in the L/R direction. Figure S5 shows similar plots along lines of voxels in the A/P direction. These spectra indicate what might be residual signal in the metabolite spectral range that might arise from lipids in the skull in the periphery of the brain.

The in vivo temperature maps using the NAA peak as reference from 6 slices of a 3D GREPSI acquisition of the healthy volunteer are shown in Figure 6. The top row shows the MPRAGE images, the middle row the temperature maps for the corresponding slices and the bottom row shows the temperatures overlaid on the anatomical MPRAGE magnitude images. With 8 slices across the 40 mm slab volume, the voxels are significantly smaller than used in the phantom study (0.112 cm^3^ vs. 0.446 cm^3^). Figure S6 shows temperature maps obtained using NAA, Cre, and Cho as references. In this case these temperatures used the slopes of Maudsley with offsets changed by +1.5°C (NAA), 0°C (Cre) and −1.0°C (Cho). (Maudsley's offsets were: 310.5°C, 206.1°C, and 187.7°C respectively).

Finally, in Figure 7 we show the results of 6 sequential 3D GREPSI temperature acquisitions on the human volunteer using the same GREPSI protocol as used for the measurements in Figure 6. Some difference in the temperature distribution can be seen in Figure 7A. The corresponding MPRAGE images are given in the top row of part B, with temperatures from the 4th time overlayed in the 2nd row of part B. The standard deviation for the left four slices is < 0.3°C (bottom row). Finally, a single CSI image was acquired covering a single slice near the center of the GREPSI images (see the MPRAGE images in part B). Acquisition times were about 8:20 for the 6 GREPSI repetitions (about 75 s each repetition) and over 12 min for the single slice 32 × 32 CSI acquisition.

## 4 Discussion

This study has presented a unique method to measure the absolute temperature in the brain using a gradient echo echoplanar spectroscopic imaging (GREPSI) sequence and a post processing method that overcomes many challenges of gradient echo spectroscopy. By eliminating refocusing and saturation pulses, GREPSI obtains increased SNR at much shorter repetition times. In scans of a standard commercially available brain metabolite phantom, using NAA as the reference peak, the method achieved a temperature precision on the order of 0.2°C for strong and weak water suppression and just over 0.3°C with no water suppression. The dependence of the measured spectra on temperature was assessed with measurements made after holding the phantom at a desired temperature for at least 24 h before each measurement. A published relationship was used for converting the peak spacing to temperature, and these studies resulted in < 1°C deviation from the independently measured phantom temperature.

GREPSI achieved rapid acquisition by eliminating refocusing and water saturation pulses and using a short (385 ms) TR. With no water suppression, the dispersive component of the water signal at the resonant frequency of the metabolite was more than an order of magnitude greater than the metabolite peak, making metabolite peak recognition and linear phase correction difficult. Because only the amplitude and not the phase of the water signal varied with the FID delay time, delaying the FID start time was able to separate the water signal from the metabolite signal. The FID delay was accomplished by simply shifting the half‐Hann window in time to start on subsequent gradient lobes in the echo train, yielding a set of spectra in which the phase of the metabolite peak relative to the water peak changes as a function of this delay. Simply averaging the set of time shifted spectra results in near complete suppression of the signals from the metabolites, leaving a reasonable estimate of the water spectrum. Subtracting this estimate of the water spectrum from the original spectra shows the metabolite peaks rotating around a very small signal. After using a low order least squares fit to remove any residual water signal, it was possible to use a linear phase correction to obtain the real spectra for the metabolites.

As shown in Figure 2, this processing method yields metabolite peaks that are nearly independent of whether water suppression was performed or not. For the phantom study, it was generally observed that the lack of water suppression reduced the precision of the resulting measurement, increasing the standard deviation from about 0.2°C to slightly over 0.3°C. Interestingly, in the one human brain study, there was some interfering fat signal near the skull, and having a strong water signal with no water suppression ensured that initial phase correction to the water signal was accurately performed. Note that neither spatial nor spectral suppression of the fat signal was performed. In this study, there are regions of apparent temperature variation that are beyond the expected noise variation, and the areas of apparent increased temperature relative to surrounding brain appear to be in periventricular white matter. Given that this is a single observation, the significance of the apparent warm points is unknown. However similar distributions were observed in the repeated acquisitions of Figure 7, which were acquired several months after the measurements of Figure 6. but the observation suggests that useful information can be obtained using this technique.

Our calibration study (Figure 4) validated the method across a physiologically relevant temperature range (22°C to 42°C). The derived *R* ^2^ values exceeding 0.99, slopes near unity, and offsets near zero confirm that temperatures measured using the slopes and offsets published by Maudsley [50], with a slight change to the offset, are accurate and that this method is robust against B_0_ field drift over several months.

### 4.1 Limitations

Although encouraging, the study has several limitations. Nearly all processing methods were developed and tested on a phantom containing water and metabolites and a single human volunteer. Note that the temperature range tested was from room temperature of about 21°C to 42°C. We did not test beyond that range out of concern to avoid damaging metabolites in the phantom itself. Calibration over higher temperatures remains to be done. The human volunteer studies presented are very promising. While out‐of‐volume saturation pulses were omitted to improve sequence efficiency, significant lipid contamination was not observed within the brain parenchyma. As indicated in Figures 5B and S5, lipid signals remained localized to the skull marrow and skin, having negligible impact on the spectral regions of interest for the metabolites. In this case, no water suppression was used to avoid excessively strong fat signal relative to the water signal. More human studies will be needed to confirm that the method can provide accurate and reliable peak locations in the presence of surrounding skull and lipid tissues.

The present study is best understood as a proof‐of‐concept, establishing the methodological foundation for multi‐subject validation in future work. Direct verification of lipid signal contamination of the metabolite peaks was not performed in this study; further validation in subjects with larger lipid deposits, such as increased subcutaneous fat or pathological lipid accumulation in brain parenchyma, will be needed to confirm that lipid contamination does not introduce systematic error in the temperature estimates, particularly in lateral brain regions near the skull. While eddy‐current‐induced water sidebands were not observed in our data—consistent with the well‐characterized eddy current compensation of the Siemens Prisma gradient system—such sidebands represent a potential source of artifact in gradient echo spectroscopic acquisitions on other hardware, and their impact on FID‐shifting‐based water suppression should be characterized in future work.

Because of the short spectral readout time (323 ms), the spectra obtained are inferior to the spectral quality that could be obtained with alternative state‐of‐the‐art spin‐echo based spectroscopic imaging approaches. Although it is possible that longer FID acquisition times could help identify and quantify metabolites, the intent with this work has been to demonstrate an efficient approach for volumetric spectroscopic imaging to detect peaks with sufficient accuracy and precision to perform rapid absolute temperature measurements. Because of the low spectral resolution, our methods of peak localization are very primitive. More highly developed methods of metabolite quantification and peak localization, such as the LCModel [68, 69], could be applied and could reduce uncertainty and noise in the temperature measurements.

In the present paper, we only present human results for using NAA as the metabolic reference for temperature measurement. Although we show primitive temperature images obtained using Cre and Cho in Figure S6, more work needs to be performed to ensure the accuracy and reliability of detecting Cre and Cho peaks.

No attempt was made to optimize the sequence or the processing methods to obtain maximum SNR. It is likely that the repetition time, flip angle, echo‐train length, and the fraction of the data used will vary depending on the SNR of the spectra and potentially other confounding artifacts.

The number of shifts of the Hann window to include was not further investigated. The plots in Figure 3 indicate that multiples of 3, 4, and 5 might be optimal to suppress NAA, Cre, and Cho signals, respectively. Thus, the value of 20 chosen might be reasonable for all three metabolites. Finally, averaging the Hann‐window‐shifted data does not guarantee complete removal of the metabolite signal. More robust results could be obtained using a parametric fit to the shifted measurements.

It is unknown why the temperature offset for the calibration study was slightly different than the previously published values. The need for a slight offset change is consistent with the finding of others that indicate the need for calibration to the specific MRI scanner [70]. The data for Figure 4 were obtained over several months, with no more than one data point for each method obtained on any single day. Except for the frequency offset, the multiple lines on Figure 4 demonstrate the near equivalence of our calibration slope to that published by Maudsley [50]. It will be important to perform a more careful calibration to ensure that the measured temperature is accurate to < 1°C.

Finally, the time for temperature measurement was reduced solely by changes in the pulse sequence (removing refocusing and saturation pulses, shortening readout and repetition time). Parallel imaging, partial Fourier acquisition, constrained reconstruction, and other methods could be used to further decrease the acquisition time.

## 5 Conclusions

The GREPSI acquisition method coupled with the Hann‐window shifting analysis results in spectra measurements where each metabolite peak can be accurately located. Linear phase correction after removal of the water signal results in real spectra showing the desired metabolite peaks. The temperature measurements obtained have shown high precision in terms of a very low standard deviation, almost independent of the type of water suppression used or not used.

## Funding

This work was supported by the National Institutes of Health, R37CA224141, R01EB028316, S10OD018482.

## Supporting information


**Figure S1:** Complex signal from a single central voxel at the NAA, Cre, and Cho peaks plotted as imaginary vs. real as a function of the effective signal delay from the RF excitation for strong water suppression (A), weak water suppression (B) and no water suppression (C). The top row is from the original measured spectrum at the position of the metabolite peaks. The middle row is after subtraction of the average of the spectrum, presumed to be close to the water signal value. The bottom row is after subtracting a low order (2nd order) fit to the spectrum. The point with the red asterisk is the point from the Hann window center on the first lobe with no signal delay, and the subsequent points are from application of the Hann window delayed by increments of one gradient lobe (1.12 ms).
**Figure S2:** Complex signal with weak water suppression for a single central voxel at the NAA, Cre, and Cho peaks plotted as imaginary vs. real values (o) with one point for each of the 20 shifts of the Hann window. The line is plotted to connect subsequent points. The position of each point is seen to be a function of the effective signal delay from the RF excitation. (A) Values from original spectrum; (B) Values after subtracting water signal estimate (average of the Hann window shifts); (C) Values after further subtraction of a low order (2nd order) fit to the signal. The top (bottom) row is from positive (negative) lobes. The point with the red asterisk is the point from the signal with no Hann window delay, and the subsequent points are from application of the Hann window delayed by increments of one gradient lobe (1.12 ms). For every case, the spectrum is corrected to zeroth order to the phase of the water peak.
**Figure S3:** Spectra with the 20 Hann window shifts from a single central voxel with weak water suppression covering the range of the metabolites with phase correction to the peaks of (a) water, (b) NAA, (c) creatine (Cre), (d) choline (Cho).
**Figure S4:** Temperature images of the phantom for strong‐, weak‐, and no‐ water suppression obtained from the difference of the water peak and the NAA peak (top row), Creatine peak (middle row) and Choline peak (bottom row). As indicated, the in each block the left temperatures are from the positive gradient lobes, the right images are from the negative gradient lobes and the right image is from averaging the images from the positive and negative gradient lobes.
**Figure S5:** Metabolite spectra (real channel after linear phase correction) from the 64 × 64 spectra of slice 3 of an 8 slice 3D GREPSI acquisition (same data as Figure 6). Each block gives the spectra from a line of voxels in the A/P direction. The line number in the L/R direction is given in each block (11, 14, 17, etc.).
**Figure S6:** Temperature for 6 slices of Figure 6 obtained using NAA, Cre, and Cho as metabolite reference. Temperature offsets for Cre and Cho were 207.1°C and 186.7°C. The cause of the shift between in vivo and the prior phantom studies is not known.

## Data Availability

The data that support the findings of this study are available on request from the corresponding author. The data are not publicly available due to privacy or ethical restrictions.
