# Supplementary material for: The Potential for Absolute Temperature Imaging Based on Brain Metabolites Using an FID‐Shifting Approach in Gradient Echo Planar Spectroscopic Imaging (GREPSI)
Source: Magn Reson Med. 2026 Jun 23;96(5):2044–57. doi: 10.1002/mrm.70487 (PMC13527235; doi:10.1002/mrm.70487)
Supplement: Supplementary file 1 [file MRM-96-2044-s001.docx]

The potential for absolute temperature imaging based on brain metabolites using Gradient Echo Planar Spectroscopic Imaging (GREPSI)

Dennis L. Parker, Henrik Odéen, Peyton Wong, Seong-Eun Kim, Wesley Judd, Duane D. Blatter

Affiliation:

Utah Center for Advanced Imaging Research,

Department of Radiology and Imaging Sciences

University of Utah, Salt Lake City, Utah

Corresponding Author:

Dennis L. Parker, PhD

Utah Center for Advanced Imaging Research

Department of Radiology and Imaging Sciences

University of Utah,

729 Arapeen Drive,

Salt Lake City, Utah 84128

e-mail: dennis.parker@hsc.utah.edu

To motivate the FID-shifting method used in this paper, we consider Equation 8 for a single spectral component (i.e. water or metabolite) for a single arbitrary position:

|  | $s_{l}\left( f_{k} \right)=\sum_{j=l}^{N_{Hann}} h\left( \frac{j-1}{N_{FID}} \right)m^{'}\left( t_{l}+j\Delta t \right)e^{-2\pi i\frac{kj}{N_{zfi}}}$ | (s1) |
| --- | --- | --- |

Here, $f_{k}t_{j}=k\Delta f j\Delta t=\frac{kj}{N_{zfi}}$ which implies that $\Delta f=\frac{1}{N_{zfi}\Delta t}$ . In the case of mono-exponential decay Eq. (s1) can be rewritten:

|  | $s_{l}\left( f_{k} \right)=m^{'}\left( t_{l} \right)\sum_{j=l}^{N_{Hann}+l-1} h\left( \frac{j-1}{N_{FID}} \right)e^{-\frac{j\Delta t}{T_{2}}}e^{-2\pi i\frac{kj}{N_{zfi}}}$ | (s2) |
| --- | --- | --- |

The summation in Eq. (s2) does not depend on the shift, $l$, and can be written as:

|  | $g(f_{k})=\sum_{j=l}^{N_{Hann}+l-1} h\left( \frac{j-1}{N_{FID}} \right)e^{-\frac{j\Delta t}{T_{2}}}e^{-2\pi i\frac{kj}{N_{zfi}}}$ | (s3) |
| --- | --- | --- |

For *N_FID_* sufficiently long, the summation can be approximated by the integral:

|  | $g\left( f_{k} \right)=\int_{0}^{\infty} {dt e}^{-\frac{t}{T_{2}}}e^{-2\pi ift}=\frac{T_{2}}{1+4\pi^{2}f^{2}T_{2}^{2}}-\frac{i2\pi fT_{2}^{2}}{1+4\pi^{2}f^{2}T_{2}^{2}}$ | (s4) |
| --- | --- | --- |

On resonance, when $f=0$, $g\left( 0 \right)=T_{2}$. For $f\neq0$, $g(f)$ will have a phase that depends on $f$ but not on $l$. The phase of $s_{l}\left( f_{k} \right)$ depends on $l$ through the term $m^{'}\left( t_{l} \right)$ which can be written:

|  | $m^{'}\left( t_{l} \right)=\left\vert m^{'}\left( 0 \right) \right\vert{dt e}^{-\frac{t_{l}}{T_{2}}}e^{-2\pi ift_{l}}$ | (s5) |
| --- | --- | --- |

The signal of metabolite, y, where (y = NAA, Cre, Cho, etc.) vs frequency can be written:

|  | $s_{y,l}(f)=\left\vert m_{y}^{'}\left( 0 \right) \right\vert{dt e}^{-\frac{t_{l}}{T_{2,y}}}e^{-2\pi if_{y}t_{l}} g(f)$ | (s6) |
| --- | --- | --- |

Likewise, the water signal can be written

|  | $s_{w,l}(f)=\left\vert m_{w}^{'}\left( 0 \right) \right\vert{dt e}^{-\frac{t_{l}}{T_{2,w}}}e^{-2\pi if_{w}t_{l}} g(f)$ | (s7) |
| --- | --- | --- |

Finally, the phases of the metabolite signals at the metabolite resonance can be obtained:

|  | $s_{y,l}(f_{y})=\left\vert m_{y}^{'}\left( 0 \right) \right\vert{dt e}^{-\frac{t_{l}}{T_{2,y}}}e^{-2\pi if_{y}t_{l}} T_{2,y}$ | (s8) |
| --- | --- | --- |

giving the phase:

|  | $\varphi_{y,l}(f_{y})=-2\pi f_{y}t_{l}$ | (s9) |
| --- | --- | --- |

The phase of the water signal at the same frequency can obtained from:

|  | $s_{w,l}(f)=\left\vert m_{w}^{'}\left( 0 \right) \right\vert{dt e}^{-\frac{t_{l}}{T_{2,w}}}e^{-2\pi if_{w}t_{l}} g(f_{NAA})$ | (s10) |
| --- | --- | --- |

giving the phase:

|  | $\varphi_{w,l}(f_{y})=-2\pi f_{w}t_{l}+\varphi_{g}$ | (s11) |
| --- | --- | --- |

Note that $\varphi_{g}$ does not depend on the FID shift, $l$. Thus, the phase difference between the water and metabolite signals at the metabolite reference frequency changes linearly with $t_{l}$:

|  | $\varphi_{w,l}\left( f_{y} \right)-\varphi_{y,l}(f_{y})=2\pi\left( f_{y}-f_{w} \right)t_{l}+\varphi_{g}$ | (s12) |
| --- | --- | --- |

Figure S1: Complex signal from a single central voxel at the NAA, Cre, and Cho peaks plotted as imaginary vs. real as a function of the effective signal delay from the RF excitation for strong water suppression (A), weak water suppression (B) and no water suppression (C). The top row is from the original measured spectrum at the position of the metabolite peaks. The middle row is after subtraction of the average of the spectrum, presumed to be close to the water signal value. The bottom row is after subtracting a low order (2^nd^ order) fit to the spectrum. The point with the red asterisk is the point from the Hann window center on the first lobe with no signal delay, and the subsequent points are from application of the Hann window delayed by increments of one gradient lobe (1.12 ms).

Figure S2: Complex signal with weak water suppression for a single central voxel at the NAA, Cre, and Cho peaks plotted as imaginary vs. real values (o) with one point for each of the 20 shifts of the Hann window. The line is plotted to connect subsequent points. The position of each point is seen to be a function of the effective signal delay from the RF excitation. A) Values from original spectrum; B) Values after subtracting water signal estimate (average of the Hann window shifts); C) Values after further subtraction of a low order (2^nd^ order) fit to the signal. The top (bottom) row is from positive (negative) lobes. The point with the red asterisk is the point from the signal with no Hann window delay, and the subsequent points are from application of the Hann window delayed by increments of one gradient lobe (1.12 ms). For every case, the spectrum is corrected to zeroth order to the phase of the water peak.

Figure S3: Spectra with the 20 Hann window shifts from a single central voxel with weak water suppression covering the range of the metabolites with phase correction to the peaks of (a) water, (b) NAA, (c) creatine (Cre), (d) choline (Cho)

Figure S4: Temperature images of the phantom for strong-, weak-, and no- water suppression obtained from the difference of the water peak and the NAA peak (top row), Creatine peak (middle row) and Choline peak (bottom row). As indicated, the in each block the left temperatures are from the positive gradient lobes, the right images are from the negative gradient lobes and the right image is from averaging the images from the positive and negative gradient lobes.

**Figure S5**: Metabolite spectra (real channel after linear phase correction) from the 64x64 spectra of slice 3 of an 8 slice 3D GREPSI acquisition (same data as Figure 6). Each block gives the spectra from a line of voxels in the A/P direction. The line number in the L/R direction is given in each block (11, 14, 17, etc.).

Figure S6: Temperature for 6 slices of Figure 6 obtained using NAA, Cre, and Cho as metabolite reference. Temperature offsets for Cre and Cho were 207.1 and 186.7 °C. The cause of the shift between in vivo and the prior phantom studies is not known.
